# Dopamine Neurons in the Ventral Tegmental Area: An Autopsy Case of Disorganized Type of Schizophrenia

**DOI:** 10.1155/2011/381059

**Published:** 2011-09-22

**Authors:** Keiko Ikemoto, Tatsuro Oda, Akiyoshi Nishimura, Katsuji Nishi

**Affiliations:** ^1^Department of Neuropsychiatry, School of Medicine, Fukushima Medical University, 1 Hikarigaoka, Fukushima 960-1295, Japan; ^2^Department of Psychiatry, Iwaki Kyoritsu General Hospital, Iwaki 973-8555, Japan; ^3^Department of Legal Medicine, Shiga University of Medical Science, Otsu 520-2192, Japan; ^4^Department of Psychiatry, National Hospital Organization Shimofusa Psychiatric Medical Center, Chiba 266-0007, Japan; ^5^Department of Forensic Medicine, Institute of Health Bioscience, Tokushima University Graduate School, Tokushima 770-8503, Japan

## Abstract

The mesolimbic dopamine (DA) system has been associated with the pathogenesis of schizophrenia. Here, we examined DA-containing neuronal structures of the ventral tegmental area (VTA) of an autopsy case of disorganized type of schizophrenia (75-year-old female), using tyrosine hydroxylase (TH) immunohistochemistry. A free floating method using 50-**μ**m cryostat sections and three-dimensional imaging analyzer AxioVision were applied to observe the wide range structures of TH-immunoreactive (-ir) neurons. TH-ir neuronal cell bodies in the VTA of the present case had irregular shape and various size, and TH-ir neuronal processes had irregular thickness and straightened shape or curved shape having many corners, when compared to a control autopsy case with no detectable neurological and psychiatric diseases (64-year-old male). The mechanisms underlying the morphological characteristics of DA neurons of the brains with schizophrenia should be elucidated epigenetically as well as genetically.

## 1. Introduction

Dopamine (DA) dysfunction [1–3], glutamate dysfunction [4, 5], or neurodevelopmental deficits [6] are widely accepted hypotheses for etiology of schizophrenia. The mesolimbic DA system has been a major interest of schizophrenia study [1, 3, 7], because this neuronal system, originating from the ventral tegmental area (VTA, A10) to the nucleus accumbens (Acc), known for an antipsychotic acting site [8, 9], is involved in motivation, emotion, reward, and learning and is involved in the pathogenesis of drug dependence [10] and schizophrenia [7, 11]. The VTA receives fiber projections from the Acc, ventral pallidum, amygdala, lateral habenular nucleus, laterodorsal tegmental nucleus, dorsal raphe nucleus, locus coeruleus, and lateral hypothalamus [12]. 

Recent histopathological studies using schizophrenic postmortem brains showed minor deficits of neural networks [13] mainly limited in the dorsolateral prefrontal cortex [14–16], anterior cingulate cortex [17], entorhinal cortex [16, 18, 19], and hippocampus [20, 21]. Nevertheless, there have been only few morphological studies on midbrain DA neurons of patients with schizophrenia. Bogerts et al. [22] reported that mean volume of the nerve cells is diminished in the VTA of drug-naive schizophrenia [22], and concordantly Nopoulos et al. [23] showed the volume reduction of the midbrain of patients with schizophrenia using MRI imaging [23].

Here, the authors report the morphological characteristics of DA neurons in the VTA of an autopsy case of disorganized type of schizophrenia [1–3, 7]. Tyrosine hydroxylase (TH), the first-step synthesizing enzyme for catecholamines, has been used as a marker of midbarin DA neurons [24–26], and TH-containing neuronal structures including neural processes have been observed using an image analyzer AxioVision [9, 27].

## 2. Materials and Methods

### 2.1. Autopsy Cases

The case was a 75-year old female, diagnosed as disorganized type of schizophrenia, using the Diagnostic Criteria from DSM-IV. The duration of illness was approximately 50 years. During one month preceding the death, no neuroleptics have been prescribed. The postmortem brain was obtained by a pathological autopsy (post-mortem interval (PMI): 8 hours) in the National Hospital Organization, Shimofusa Psychiatric Center, Japan with approval of the Ethical Committee of National Hospital Organization, Shimofusa Psychiatric Center. 

As a control case, the legal autopsy case of a 64-year-old male was used. The autopsy was performed in the Department of Legal Medicine, Shiga University of Medical Science, Japan, in compliance with the ethical codes of the Ethical Committee of the Japanese Society of Legal Medicine, and the Brain Bank of Shiga University of Medical Science. The cause of death was acute myocardial infarct (PMI: 4 hours), and the case had no known clinically and pathologically detectable neurological and psychiatric diseases.

### 2.2. Tissue Preparations

Brains were immediately sliced into 1 cm slabs and immersed in the fresh fixative at 4°C for 48–72 hours. Tissue preparations were performed based on previous studies [28]. The slices were then transferred to phosphate buffer containing 15% sucrose and 0.1% sodium azide for storage at 4°C. The brain sections were cut using a cryostat in 50 *μ*m thick in coronal planes through the midbrain, in order to observe the wide range structures of dendrites and the axons. The sections were treated with 40% methanol and 1% H_2_O_2_ for 20 minutes to inhibit endogenous peroxidase [28].

### 2.3. Immunocytochemistry

For TH immunocytochemistry, rabbit anti-TH antibody [27] diluted 1 : 10,000–30,000 in 0.1 M phosphate-buffered saline containing 0.3% Triton X-100 were used. Free floating method and ABC-DAB method were applied. Details of immunocytochemical procedures for TH were also described previously [28]. At least three sections were stained for each brain.

### 2.4. Data Analysis

The structures of TH-positive neurons were observed under light microscope. The images of focuses at 13~15 levels per a 50 *μ*m thick section were three dimensionally reconstructed by using the image analyzer, all in Focus in AxioVision system (Zeiss, Germany). The detailed morphological characteristics of TH-ir neuronal cell bodies and neural processes were observed. An atlas of Mai et al. [29] was used to identify the anatomical territories [29]. 

## 3. Results

**Figure 1:**
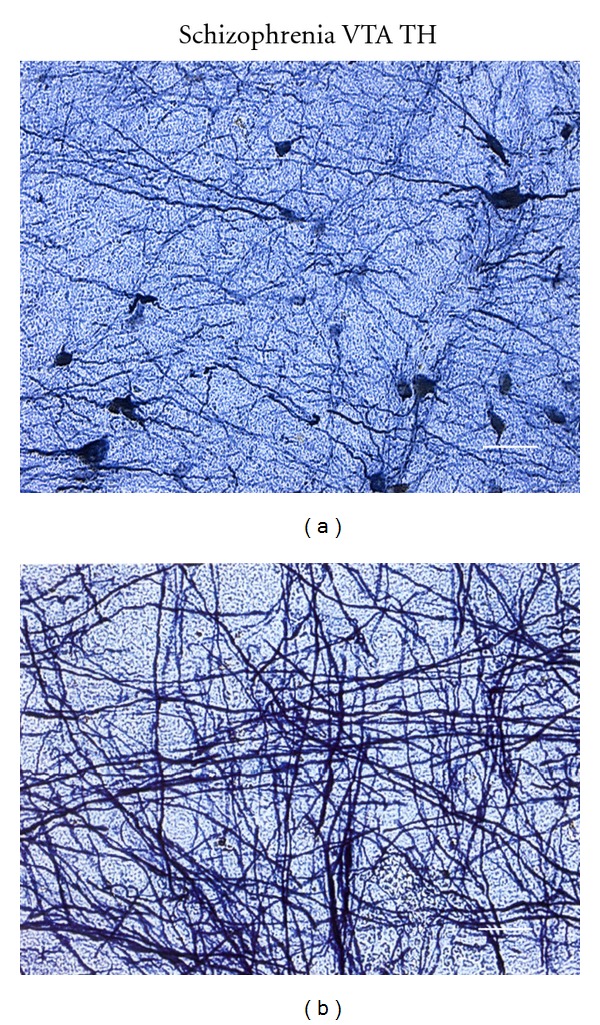
An image synthesized by AxioVision from a 50 *μ*m thick TH-stained section of the ventral tegmental area (VTA) of a 75-year-old female patient with disorganized type of schizophrenia from a pathological autopsy. TH-ir fibers were magnifies in (b)/(a,b) TH-ir neuronal cell bodies have irregular shape, including multipolar-, triangular- or oval-shaped, and various size (6–24 *μ*m in diameter) (Figure 1(a)). The stainability varies between the neuronal cell bodies. TH-ir neuronal processes had irregular thickness (Figure 1(a)), curved shape having many corners (Figure 1(a)), straightened (Figure 1(b)), or are composed of fiber bundles (Figure 1(b)). These morphological findings of TH-ir neurons in the VTA were also seen in the substantia nigra of schizophrenia (not shown). Bars: Figure 1(a): 25 *μ*m, Figure 1(b): 12.5 *μ*m.

In the brain of the case with clinically diagnosed disorganized type of schizophrenia, TH-immunoreactive (-ir) neuronal cell bodies in the VTA had irregular shape, including multipolar-, triangular- or oval-shaped neurons, and various size (6–24 *μ*m in diameter) (Figure 1(a)), and the stainability varied between the neuronal cell bodies (Figure 1(a)). TH-ir neuronal processes had irregular thickness (Figure 1(a)), curved shape having many corners (Figure 1(a)), straightened (Figure 1(b)), or were composed of fiber bundles (Figure 1(b)). These morphological findings of TH-ir neurons in the VTA were also seen in the substantia nigra (not shown).

**Figure 2:**
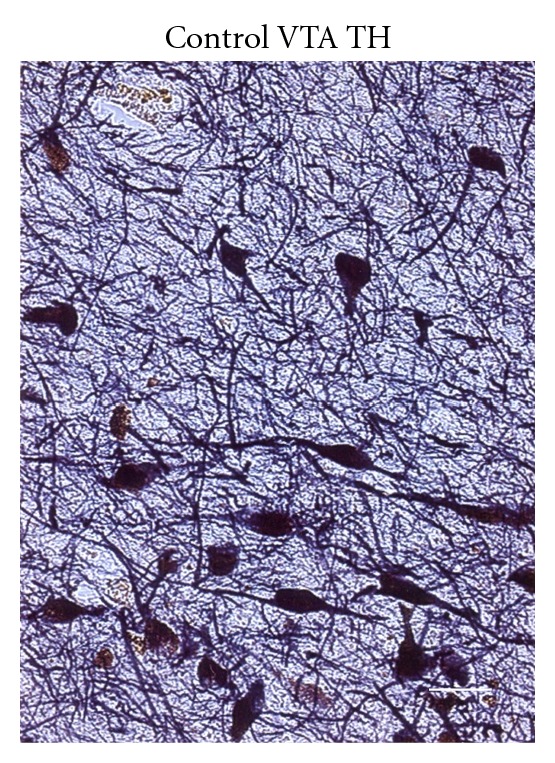
An image synthesized by AxioVision from a 50 *μ*m thick TH-stained section of the ventral tegmental area (VTA) of a 64-year-old male case of a legal autopsy. The VTA contains TH-ir neuronal cell bodies with diameter of 12–26 *μ*m. The size of these neurons is comparatively similar. The shape of TH-ir neuronal cell bodies is bipolar, fusiform, oval or triangular and is distinct from that of the case with disorganized type, schizophrenia (Figure 1(a)). Bar: 25 *μ*m.

In the control case, the VTA contained TH-ir neuronal cell bodies with diameter of 12–26 *μ*m. The size of these neurons was comparatively similar as shown in Figure 2. The shape of TH-ir neuronal cell bodies was bipolar, fusiform, oval, or triangular and was distinct from that of the case with disorganized type of schizophrenia. TH-ir processes of the TH-ir neurons had many varicosities (not shown). We could not notice TH-ir fiber bundles or straightened-shaped TH-ir neural processes in the control case (Figure 2).

## 4. Discussion

The present study demonstrated several morphological characteristics of TH-ir neurons in the VTA of an autopsy case with disorganized type of schizophrenia.

The previous morphological studies showed hypoplastic midbrain [23] and reduced number and small size of midbrain DA neurons in drug-naïve patients with schizophrenia [22]. The result in the present study also showed irregular (and putatively smaller) sized midbrain DA neurons. In addition, we also described the morphological characteristics of DA fibers, having corners, irregular thickness or being straight shaped. 

The present methods of a free floating method using 50 *μ*m thick sections [25, 30] and three-dimensional reconstruction of TH-ir fibers using AxioVision (Zeiss, Germany) were much better than usual paraffin sections at the thickness of 3–5 *μ*m, to observe wide ranges of TH-ir structures, including TH-ir neural processes. However, due to complexity of experimental procedures and difficulties in obtaining qualified autopsy brains in Japan, we failed to perform detailed statistical analysis. Based on a single case, it is unknown if similar changes in the VTA can be found in schizophrenia in general and might be specific to a small subset of patients. 

A major question arising from the present results is whether DA neuronal structures found in the VTA of the case with schizophrenia were effects of long-term administration of typical antipsychotic drugs. In order to confirm that, we preliminary administrated haloperidol to Wistar rats for seven months and observed no similar morphological changes to the present results (unpublished data). 

Recent genetic studies showed that schizophrenia susceptible genes including DISC1 relate to neural development or synapse formation [31]. The integrative nuclear fibroblast growth factor receptor-1 (FGFR1) knockout mice, showing schizophrenia-like behavior including reduced prepulse inhibition, displayed recovery from these behavioral deficits by antipsychotic administration, and the size and quantity of midbrain DA cells of the model mice showed significant reduction [32].

Further studies should be conducted to examine the mechanisms of genetic regulations and/or environmental influences that produce the characteristic morphological findings of DA neurons shown in the present study.
